# Down‐regulated lncRNA AGAP2‐AS1 contributes to pre‐eclampsia as a competing endogenous RNA for JDP2 by impairing trophoblastic phenotype

**DOI:** 10.1111/jcmm.15113

**Published:** 2020-03-09

**Authors:** Yetao Xu, Xi Xia, Ying Jiang, Dan Wu, Sailan Wang, Shilong Fu, Nana Yang, Yuanyuan Zhang, Lizhou Sun

**Affiliations:** ^1^ Department of Obstetrics and Gynecology First Affiliated Hospital of Nanjing Medical University Nanjing China; ^2^ Department of Obstetrics and Gynecology Center for Reproductive Medicine Peking University Shenzhen Hospital Shenzhen China; ^3^ Department of Obstetrics Women's Hospital Zhejiang University School of Medicine Hangzhou China

**Keywords:** AGAP2‐AS1, FOXP1, JDP2, pre‐eclampsia, trophoblastic phenotype

## Abstract

Recently, growing evidence has shown that aberrant long non‐coding RNA (lncRNA) expression in conjunction with an impaired trophoblastic phenotype could implicate the pathological process of pre‐eclampsia (PE). However, only a small portion of lncRNAs has been characterized with regard to the function and molecular mechanisms involved in PE. There are still gaps in the available knowledge; as a result, there are currently only a few applicable treatments for PE in the context of lncRNA. Here, we found that lncRNA AGAP2‐AS1 is abnormally down‐regulated in severe PE placenta tissues. Using human trophoblasts, we established that AGAP2‐AS1 knockdown could inhibit trophoblasts proliferation and invasion and promote cell apoptosis. Further, we showed that overexpression of AGAP2‐AS1 substantially stimulated the development of the trophoblastic phenotype. Through high‐throughput sequencing analysis, we demonstrated that silencing of AGAP2‐AS1 favourably regulated various genes which are relevant to trophoblastic growth and invasion. Mechanistically, AGAP2‐AS1 promoted the suppressor protein, Jun dimerization protein 2 (JDP2), by sponging miR‐574‐5p. Resultantly, further impairment of the trophoblastic phenotype was achieved by way of inhibiting cell growth, apoptosis and invasion. We also determined that the expression of AGAP2‐AS1 could be mediated by FOXP1. Our results showed that the down‐regulated expression of lncRNA AGAP2‐AS1 might serve as a key suppressor in PE via inhibition of JDP2 at the post‐transcriptional level by competing for miR‐574; thus, this presents a novel therapeutic strategy for PE.

## 1 INTRODUCTION

Pre‐eclampsia (PE) is a distinct hypertensive pregnancy disorder that occurs in approximately 5‐8% of females worldwide, and the incidence of PE has gradually increased.^1^ Around 10% of all severe cases of PE cases occur before 34 weeks of gestation. Currently, PE is considered to be a multisystem syndrome of human gestation and is related to high maternal and infant morbidity and mortality rates.^2^, ^3^ Pregnancy predisposes females to a higher risk of pulmonary oedema, hepatic and renal failure, seizures, cerebral haemorrhage and even death.^4^ Additionally, PE can impact the overall health and well‐being of the foetus. Foetal growth restriction is a common complication associated with PE, and it is considered to occur as a result of chronic placental hypoperfusion. Placental abruption and premature separation of the placenta with the disruption of blood flow to the foetus are dangerous, yet uncommon, complications that occur in up to 3% of pregnancies affected by severe PE.^5^ At present, the specific causes of PE in pregnancy remain unclear; moreover, no accurate method is available to prevent the development of PE. Thus, there is an imperative need for the development of strategies that enable the safe delay of the delivery of babies to decrease the maternal and neonatal risks associated with premature birth.

Less than 2% of the mammalian genome includes protein‐coding genes, about 90% of the genome accounts for lncRNAs 6 and the latter are transcribed rather than encoded proteins. Despite the lack of a general consensus regarding their biological characteristics, there is growing evidence that lncRNAs have a high degree of regulation and important functions in human diseases. Specifically, lncRNAs have emerged as key molecular players in the regulation of gene expression in different biological processes such as parental imprinting, stem cell pluripotency, cell growth,^7^, ^8^ and metastasis and apoptosis.^9^, ^10^ It is considered that these lncRNAs are regulated through effects such as miRNA sponging, chromatin remodelling and epigenetic modification.^11^ It is interesting to note that recently, an increasing number of studies have validated the fact that lncRNAs might be associated with diverse human pregnancy‐related disorders, especially PE.^12^, ^13^, ^14^

AGAP2‐AS1, a 1567 nt lncRNA located on chromosome 12q14.1, has been found to be abnormally expressed in multiple disease states, including cancer. Recent evidence shows that AGAP2‐AS1 could epigenetically regulate MyD88 expression to promote chemoresistance in patients with breast cancer.^15^ In addition, Li et al^16^ reported that the elevated expression of AGAP2‐AS1 regulates downstream target transcription in non–small‐cell lung cancer by interacting with epigenetic proteins. Thus, in this study, we investigated the role of AGAP2‐AS1 in the biological mechanism of PE as, at present, its role remains unclear.

In this report, through qRT‐PCR in PE placenta tissues, we found that the RNA levels of AGAP2‐AS1 were abnormally down‐regulated, further suggesting that it may be connected to the pathogenesis of PE. Subsequently, we investigated the trophoblastic phenotype, including cell growth, apoptosis and invasion, after knockdown or overexpression of AGAP2‐AS1 in vitro. Lastly, a biological mechanistic explanation was proposed as a result of our attempts to establish how AGAP2‐AS1 regulates its prospective upstream/downstream transcription of genes.

## 2 MATERIALS AND METHODS

### 2.1 Ethics statement

Human pre‐eclampsia placental tissues and normal placental samples were collected from the department of Obstetrics at Jiangsu Province Hospital, Jiangsu, China. The study was authorized by the hospital ethics board. Written informed consent was obtained from patients who met the inclusion criteria. The clinicopathological characteristics of each participant are summarized in Table 1.

**Table 1:** Clinical features of normal and PE pregnancies

Variable	PE (N = 20)	Normal (N = 20)	*P* values Normal vs *P*
Maternal age (year)	32.3 ± 3.551	34.45 ± 2.202	>.05
Maternal weight (kg)	74.875 ± 12.995	74.65 ± 7.611	>.05
Smoking	0	0	>.05
Systolic blood pressure (mm Hg)	158.5 ± 12.631	119.25 ± 7.739	<.01
Diastolic blood pressure (mm Hg)	104.4 ± 5.774	73.22 ± 7.469	<.01
Proteinuria (g/d)	>0.3 g	<0.3 g	<.05
Body weight of infant (g)	2448.5 ± 780.124	3332.5 ± 416.316	<.05
Gestational age (week)	34.05 ± 2.539	38.5 ± 0.806	<.05

### 2.2 Cell culture

HTR‐8/SVneo was generous provided by Prof. Charies Graham from Queen's University in Canada. JEG3 was from the Institute of the Chinese Academy of Sciences (Shanghai, China). JEG3 and HTR‐8/SVneo are incubated in RPMI Medium 1640 (REF: C11875500B, LOT: 8118016) and MEM (Gibco), respectively, in which added with 10% foetal bovine serum (Invitrogen) in a humidified incubator at 37°C with 5% CO**_2_**.

### 2.3 Transfections of cell lines

Suspend transfection for HTR‐8/SVneo cells: 1‐2 μL Lipofectamine 3000 were thoroughly mixed with 25 μL OPTI‐MEM. In parallel, 20pmol of siRNAs were mixed with 25 μL OPTI‐MEM through pipette. After incubation at room temperature for 5 minutes, the corresponding tubes (contained siRNAs and Lipo3000) were thoroughly mixed through moderate pipetting and then incubated 5‐10 minutes at room temperature. At the end of incubation, the 50 μL transfection solution was used to re‐suspend cell pellet (6 × 10^4^ cells/each 24‐well scale). After incubated 10 minutes at room temperature, 450 μL fresh complement medium was added to each tube and then the suspended cells were pipetted onto the culture plate.

On‐plate transfections for primary trophoblast cells: 6 × 10^4^ cells were plated on the 6‐well plate scale. After on‐plated 6 hours of primary cells, we performed the on‐plated transfection assays. 100 pmol (10 μL) siRNAs were mixed with 240 μL OPTI‐MEM by gentle pipetting and incubation for 5min. At the same time, 10 μL Lipo3000 was thoroughly mixed with 240 μL OPTI‐MEM by pipette and also incubated 5 minutes at room temperature. After incubation for 5 minutes, the corresponding tubes (contained siRNAs and Lipofectamine 3000) were mixed by moderate pipetting and then incubation for 20 minutes at room temperature. At the end of 20 minutes, 500 μL total solution was added into each 6‐well plate which have removed the medium. After 6 hours of transfection, the transfected solution was replaced by the complement medium. 40 nmol/L of miR‐574 inhibitor, miR‐574 mimic and the controls (RiboBio Co. Ltd, Guangzhou, China) were transfected into JEG3 and HTR‐8/SVneo, and the cells were incubated for 48 hours after transfection (6‐well plate scale). 4 μg plasmid (vector, pcDNA‐AGAP2‐AS1 and pcDNA‐JDP2) were used to transfect in each 6‐well plate, according to the manual.

### 2.4 Recombinant plasmid construction

To ectopically up‐regulate the expression of AGAP2‐AS1, the AGAP2‐AS1 plasmid (1567 bp, NR_027032.1, synthesized by Realgene, Nanjing, China) was subcloned into a pcDNA3.1(+) vector (Invitrogen), following standard protocols. AGAP2‐AS1 plasmids as well as empty vectors were transfected into HTR‐8/SVneo cells on 6‐well plates and/or 24‐well plates.

### 2.5 RNA extraction and qRT‐PCR analyses

RNA preparation and qRT‐PCR assays were performed as previously reported by Xu et al^7^ RNA (0.5‐0.8 μg) was reverse transcribed in a final volume of 20 μL using the PrimeScript RT Reagent kit (TaKaRa). SYBR Premix Ex Taq (TaKaRa) was used to examine the expression of AGAP2‐AS1 and JDP2, in accordance with the manufacturer's instructions. All data were normalized to GAPDH; the primer sequences are listed in Table S1.

### 2.6 High‐throughput sequencing analysis

High‐throughput sequencing assays were performed as previously reported by Xu et al^7^ Using human trophoblast cells, we performed an RNA‐seq experiment to establish the mRNA‐seq profiles after transfection with effective siRNAs against the AGAP2‐AS1 mRNA‐seq library, which was established for sequencing and exploiting standard Illumina protocols. Subsequently, we evaluated the pathways that were activated by AGAP2‐AS1 by analysing our data against the Gene Ontology (GO) database.

### 2.7 Cell proliferation assays

To assess the cell growth ability of HTR‐8/SVneo and JEG3 cell lines, MTT and EdU assays, respectively, were conducted. These experiments were carried out as per the method reported by Xu et al.^9^ For the MTT assay, HTR‐8/SVneo and JEG3 cells were transfected with mimics (NC or miRNA‐574‐5p and/or siRNAs, containing 3000 cells/well) and subsequently plated in 96‐well plates with five duplicates. Cell viability was measured every 24 hours, following standard protocols. The absorbance was detected at 490 nm using the ELx800 Universal Microplate Reader (BioTek). For the EdU assays, cells were plated into 24 wells with 5 × 10^4^ cells/well. We then used the 5‐ethynyl‐2‐deoxyuridine labelling/detection kit (RiboBio) to estimate cell viability, according to the manufacturer's instructions.

### 2.8 Flow cytometric analysis of cell cycle and apoptosis

These assays were conducted as previously described by Xu et al^10^ After transfecting cells with either siRNAs or the plasmid, we performed fluorescein isothiocyanate (FITC)‐annexin V and propidium iodide (PI) staining using the FITC‐Annexin V Apoptosis Detection kit (BD Biosciences), following the manufacturer's instructions. The cell cycle level was determined by propidium oxide staining using the Cycle Test Plus DNA Reagent kit (BD Biosciences), following the protocol. Data were analysed using the FACScan flow cytometer.

### 2.9 Migration and invasion assays

This experiment was carried out as previously described in Xu et al^10^ Briefly, HTR‐8/SVneo and JEG3 cells were transfected with specific siRNAs and/or plasmid; they were then re‐suspended in RPMI‐1640 medium containing 1% FBS and subsequently added to the upper chamber. The lower chamber was filled with 700 µl RPMI‐1640 (Gibco) medium containing 10% FBS. After incubation for 24 hours at 37°C, cells inside the upper chamber were removed before fixing. Cells on the bottom membrane surface were fixed with methanol and then stained with a 0.5% crystal violet solution. Five randomly dispersed fields were counted per well.

### 2.10 Fluorescence in situ hybridization

This experiment was conducted as defined earlier by Xu et al^10^ Firstly, HTR‐8/SVneo cells were plated in 6‐well plates. After 24‐48 hours, the cells were fixed with 75% ethanol for 30 minutes and then washed thrice with PBS. Subsequently, in accordance with standard protocols, further assays were conducted on the fixed trophoblasts. AGAP2‐AS1–effective probe sequences are listed in Table S1.

### 2.11 Chromatin immunoprecipitation assays

This experiment was conducted as defined earlier by Xu et al^10^ Trophoblast cells were preserved with formaldehyde and incubated for 10 minutes to generate DNA‐protein cross links. Then, cell lysates were sonicated to generate chromatin fragments of 200‐300 bp and immunoprecipitated with FOXP1 (Millipore) or IgG as the control. Precipitated chromatin DNA was recovered and analysed using qRT‐PCR.

### 2.12 Immunoblotting analysis

These assays were performed to detect protein levels as previously described in Xu et al^9^ Each sample (5‐10 µl) was loaded onto a 10% SDS gel and then analysed using Western blotting. The samples were incubated with specific antibodies (GAPDH from FOXP1 and JDP2 purchased from Proteintech) at 1:1000 concentration. GAPDH was used as a loading control. The intensity of the autoradiogram protein bands was quantified using the Quantity One software (Bio‐Rad).

### 2.13 Luciferase reporter assays

The assays were performed as previously described by Xu et al^10^, ^17^ The complementary DNA fragment containing the wild‐type and mutant JDP2 3′‐UTR was linked into the pGL3‐basic vector (Promega). HTR‐8/SVneo cells were cotransfected with either the wild‐type or mutant 3′‐UTR of the JDP2 vector, along with miR‐574 mimics or an miR‐574 inhibitor in the 24‐well plates. The luciferase activity of each group was assessed 48 hours after transfection using the Dual‐Luciferase Kit (Promega), 48 hours after transfection. The Renilla luciferase was used as the control.

### 2.14 Statistical analysis

All statistical analysis and graph formation were performed with GraphPad Prism version 7.0 (GraphPad Software) and Adobe Photoshop CC2015. The results were analysed using an unpaired two‐tailed *t* test and are presented as the mean ± standard error of the mean (SEM). Significance was noted at *P* < .01 (**), *P* < .05 (*).

## 3 RESULTS

### 3.1 AGAP2‐AS1 is down‐regulated in the PE placenta and is related to the gestational age and body weight of infants

**Figure 1:**
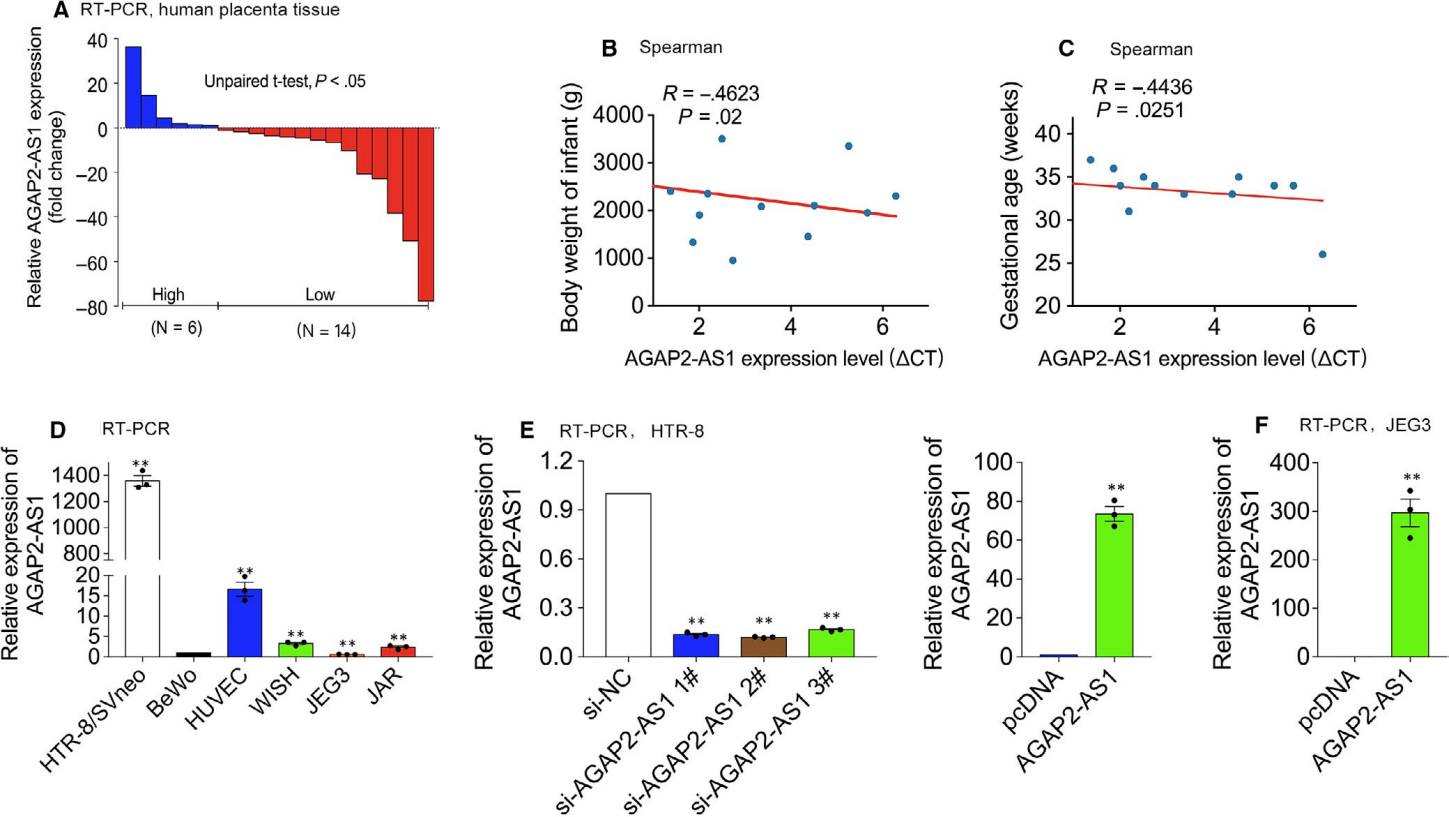
Relative AGAP2‐AS1 Expression in PE. A, qPCR results of AGAP2‐AS1 expression in pre‐eclamptic and the control (n = 20). Data are represented as log2 fold changes (PE/normal, shown as −ΔΔCT) and defined as ‘<0’ for underexpression and ‘>0’ for overexpression. The defined 0 fold of the expression represents −ΔΔCT is 0. B and C, Spearman correlations between AGAP2‐AS1 and body weight of the infant or gestational age. D, qPCR results of AGAP2‐AS1 in trophoblasts cell lines and normalized to that in BeWo. E, HTR/SVneo cells transfected with AGAP2‐AS1–specific siRNAs (siAGAP2‐AS1). F, HTR‐8/SVneo and JEG3 treated with AGAP2‐AS1 plasmid. Data are presented as the mean ± SEM, *P < .05, **P < .01

Firstly, we performed real‐time PCR to detect AGAP2‐AS1 expression in 20 normal placenta tissues and PE tissues. As shown in Figure 1A, we found that the expression of AGAP2‐AS1 was significantly decreased in the PE group, compared to that in the control group. Then, we analysed the potential correlation between the expression of AGAP2‐AS1 and the clinical features observed in patients. The clinical phenotypes are listed in Table 1. Notably, low AGAP2‐AS1 expression was significantly correlated with the gestational age (*P* < .05) and the body weight of the infants (*P* < .05). In addition, there were significant differences in the diastolic blood pressure (*P* < .01), systolic blood pressure (*P* < .01) and body weight of infants (*P* < .05), while there were no differences observed in the maternal age or maternal weight between either the PE or the control group.

### 3.2 AGAP2‐AS1 affects proliferation and apoptosis of trophoblasts

As lncRNAs could have critical functions in diverse biological processes, we suspected that the aberrant reduction of AGAP2‐AS1 in PE may affect biological processes, such as trophoblast cell migration, apoptosis and growth. Further, we postulated that these effects could be factors in the occurrence and development of PE. Thus, we explored the potential function and mechanism of AGAP2‐AS1 in trophoblasts.

**Figure 2:**
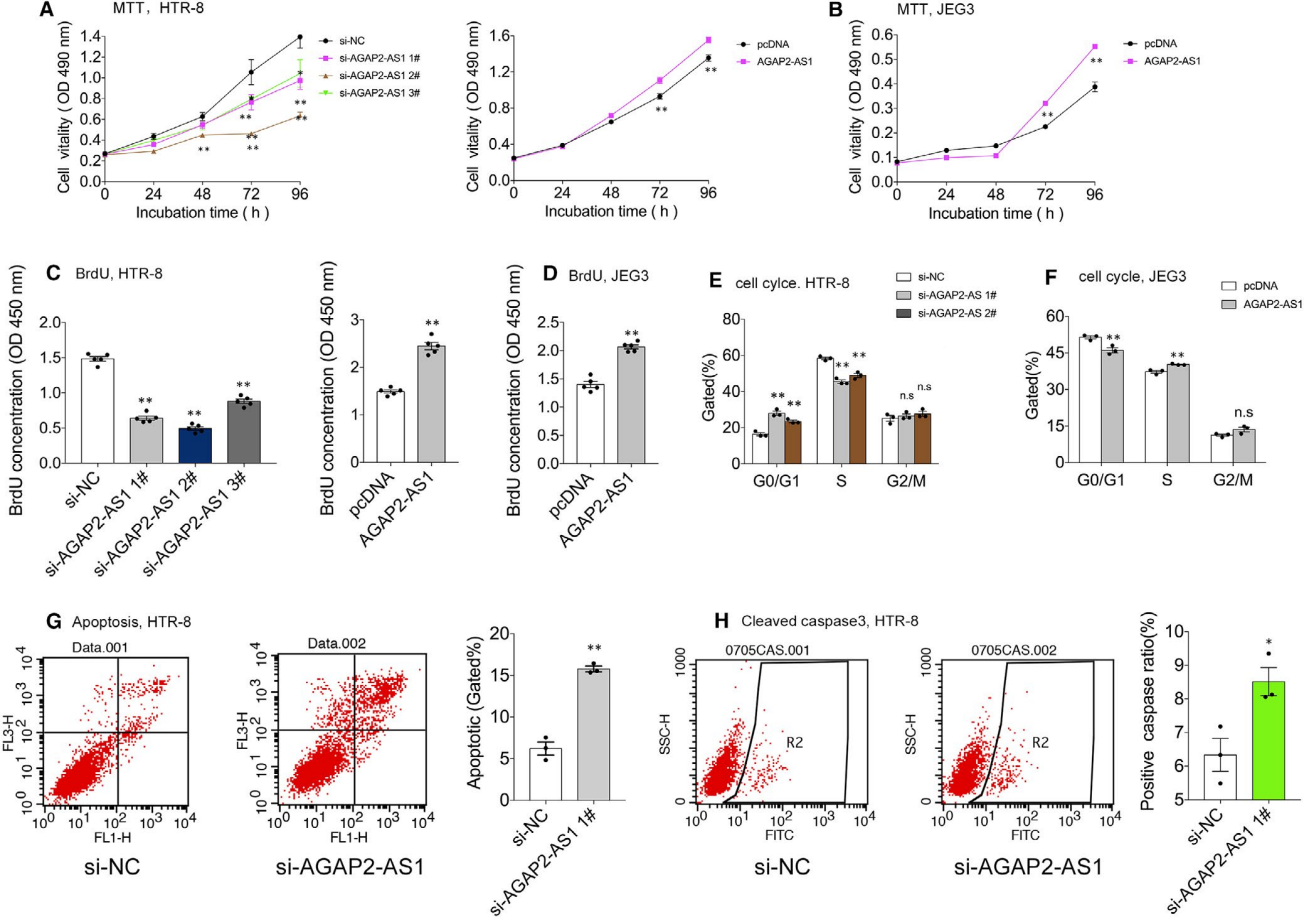
AGAP2‐AS1 affects cell proliferation and apoptosis in trophoblasts. A and B, MTT assays and (C and D) BrdU assays were conducted to test cell viability after AGAP2‐AS1 siRNAs or AGAP2‐AS1 plasmid transfected trophoblasts cells, including HTR‐8/SVneo and JEG3. E and F, Cell cycle analyses by Flow cytometry in HTR‐8/SVneo and JEG‐3 cells after transfected with AGAP2‐AS1 siRNAs or plasmid. G, Cell apoptosis analysis by Flow cytometry after transfected with AGAP2‐AS1 siRNAs in HTR‐8/SVneo. LR, early apoptotic cells; UR, terminal apoptotic cells. H, Cleaved caspase 3 protein analysis after treated with AGAP2‐AS1 siRNAs at 48 post‐transfection by Flow cytometry assays. Numbers are presented as the mean ± SEM, **P < .01, *P < .05

We tested the basic expression of AGAP2‐AS1 in trophoblast cell lines. As shown in Figure 1D, the basic expression of AGAP2‐AS1 in HTR‐8/SVneo cells was significantly higher than that in the other cell lines; however, the expression was profoundly lower in the JEG3 cell line. Thus, we selected HTR‐8/SVneo and JEG3 cells to investigate the roles of AGAP2‐AS1 in vitro. After successfully transfecting the AGAP2‐AS1–specific siRNAs, or the plasmid, in vitro, trophoblast cell viability was evaluated using the MTT and BrdU assays. When AGAP2‐AS1 was silenced (Figure 1E, left), cell growth decreased in HTR‐8/SVneo cells (Figure 2A, left; Figure 2C, left). Moreover, the elevated expression of AGAP2‐AS1 (Figure 1E, right; Figure 1F) promoted cell growth in vitro (Figure 2A, right; Figure 2B).

Next, we conducted flow cytometric analysis to examine whether down‐regulated or up‐regulated, expression of AGAP2‐AS1 can affect the cell cycle and partly reduce, or induce, cell proliferation. After the silencing of AGAP2‐AS1, significant G0/G1 phase accumulation was observed, compared to the control group (Figure 2E). Conversely, it was demonstrated that elevated expression of AGAP2‐AS1 could reduce G0/G1 phase accumulation (Figure 2F). Further, flow cytometric analysis was also performed to assess whether aberrant expression of AGAP2‐AS1 can affect trophoblast cell apoptosis. As shown in Figure 2G, the ratio of the total apoptotic cells was significantly increased after transfection with AGAP2‐AS1–specific siRNAs (siAGAP2‐AS1) in vitro. Similarly, the protein level of cleaved caspase3 was evaluated; the data showed that knockdown of AGAP2‐AS1 promoted apoptosis. Thus, these noticeable findings indicate that AGAP2‐AS1 positively affects trophoblast cell growth and, contrastingly, negatively affects cell apoptosis.

### 3.3 AGAP2‐AS1 affects migration and invasion of trophoblasts

**Figure 3:**
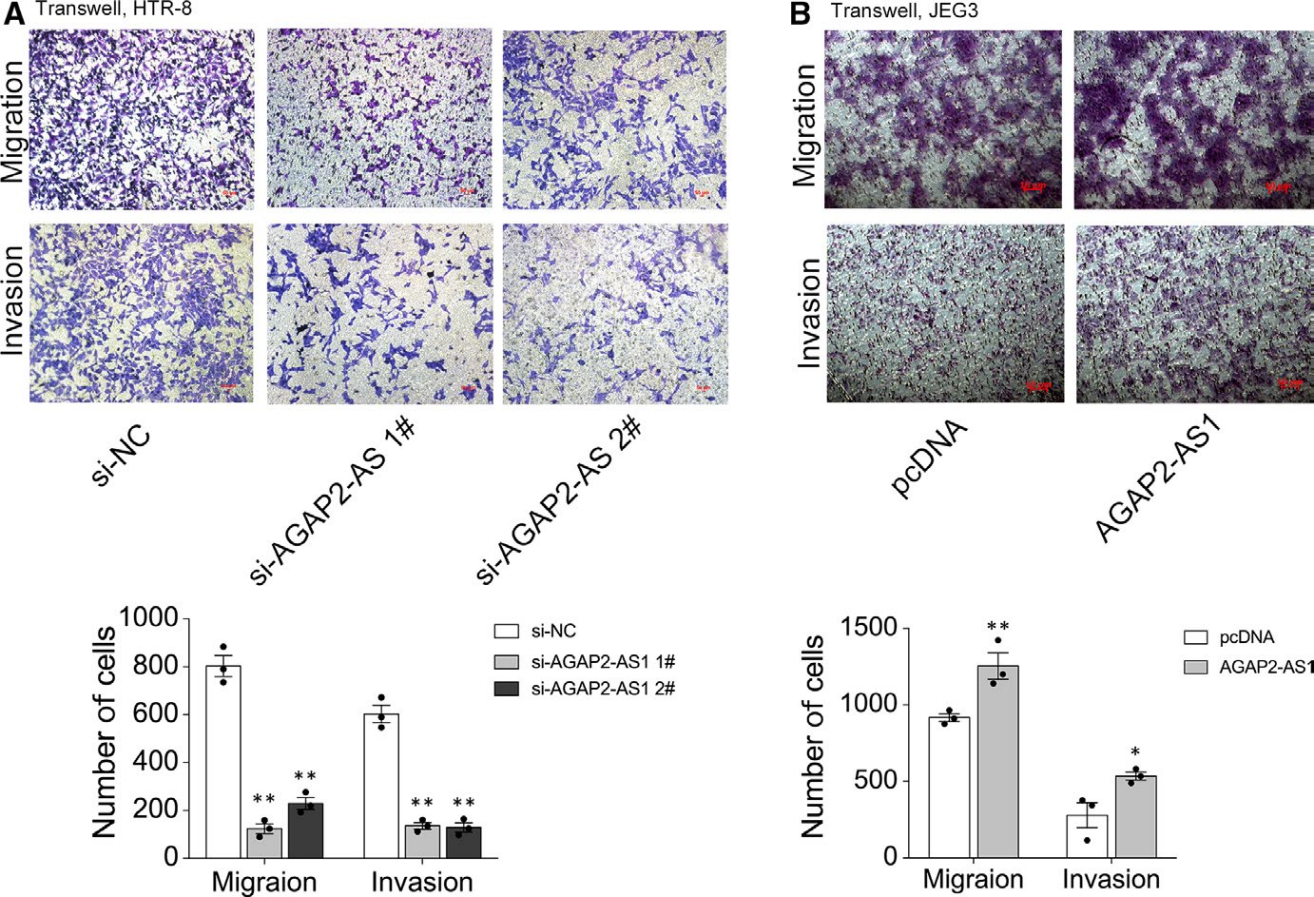
AGAP2‐AS1 affects migration and invasion of trophoblasts. A and B, Transwell assays were carried out to test migration and invasion capacity of AGAP2‐AS1 in trophoblasts. Cells on the lower chamber were stained by crystal violet and displayed, and cell number in random five perspectives was calculated. **P < .01, *P < .05

As previously noted, the abnormal ability of trophoblast migration and invasion cannot effectively induce spiral artery remodelling; moreover, its occurrence drives the progression of PE. Therefore, we performed Transwell assays to identify the effects on migration and invasion after AGAP2‐AS1 knockdown or overexpression. The results showed that the silencing of AGAP2‐AS1 in trophoblast cells can inhibit cell migration and invasion (Figure 3A). Conversely, we showed that up‐regulating the expression of AGAP2‐AS1 can induce cell migration and invasion (Figure 3B). Collectively, the data presented here highlight that AGAP2‐AS1 overexpression positively affects trophoblast cell invasion and migration.

### 3.4 AGAP2‐AS1 can be activated by the transcription factor FOXP1

**Figure 4:**
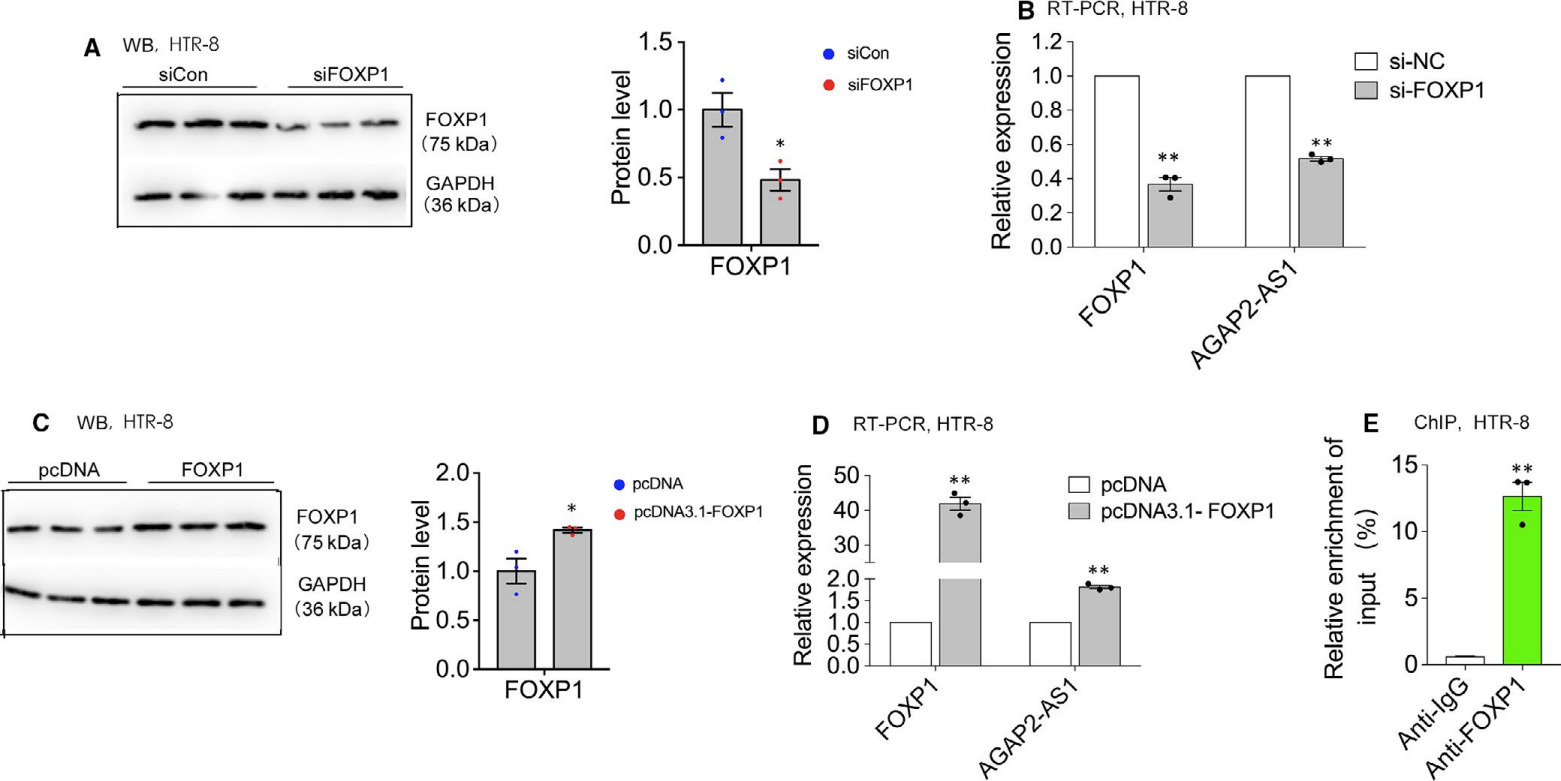
AGAP2‐AS1 can be activated by transcript factor FOXP1. A and B, FOXP1 expression level at protein and RNA level by Western blotting and qPCR, respectively, when HTR‐8/SVneo cells were transfected with FOXP1‐specific siRNAs. The AGAP2‐AS1 expression level was tested by qPCR after treated with siRNAs against AGAP2‐AS1 (B, right panels). C and D, FOXP1 expression level at protein and RNA level by Western blotting and qPCR, respectively, when HTR‐8/SVneo cells were transfected with FOXP1 plasmid. The AGAP2‐AS1 expression level was tested by qPCR after treated with plasmid (D, right panels). E, ChIP‐qPCR of FOXP1 bind to the promoter of AGAP2‐AS1 in HTR‐8/SVneo, IgG as negative control. **P < .01, *P < .05

Previous studies have reported that the majority of human lncRNA expressions can be mediated by various transcription factors.^18^, ^19^ Thus, we first used the JASPAR database (http://jaspar2014.genereg.net) to explore the latent factor that activated or inactivated the expression of AGAP2‐AS1. As shown in Table 2, 17 FOXP1‐binding sites were predicted in the promoter region of AGAP2‐AS1, which implied that AGAP2‐AS1 might be regulated by the transcription factor FOXP1 in trophoblast cells. We then used specific siRNAs and plasmids either down‐regulate or up‐regulate FOXP1 at the protein and RNA levels in the HTR‐8/SVneo cells, respectively (Figure 4A,B). The expression of AGAP2‐AS1 was significantly decreased after the silencing of FOXP1, whereas the levels of AGAP2‐AS1 were dramatically increased upon the overexpression of FOXP1 (Figure 4C,D). We then performed ChIP assays and the results indicated that transcription factor FOXP1 could bind to the promoter region to stimulate AGAP2‐AS1 expression at the RNA level (Figure 4E). The data presented here highlight that AGAP2‐AS1 can be activated by the transcription factor FOXP1.

**Table 2:** Putative FOXP1‐binding sites in the AGAP2‐AS1 promoter region by JASPAR

Model ID	Model name	Score	Relative score	Start	End	Strand	Predicted site sequence
MA0481.1	FOXP1	10.490	0.843128881436968	149	163	1	CCTGAGTAAACACTT
MA0481.1	FOXP1	11.925	0.866643541470612	537	551	1	TACAGATAAACAGAT
MA0481.1	FOXP1	9.125	0.821203714102463	1093	1107	1	AGCCAGAAAAAAAAA
MA0481.1	FOXP1	8.097	0.803915932684	1094	1108	1	GCCAGAAAAAAAAAA
MA0481.1	FOXP1	10.015	0.835345283167992	1095	1109	1	CCAGAAAAAAAAAAA
MA0481.1	FOXP1	10.996	0.851420461908762	1096	1110	1	CAGAAAAAAAAAAAA
MA0481.1	FOXP1	11.585	0.861072123762292	1097	1111	1	AGAAAAAAAAAAAAA
MA0481.1	FOXP1	11.739	0.863595648253708	1098	1112	1	GAAAAAAAAAAAAAA
MA0481.1	FOXP1	12.774	0.88055569921874	1099	1113	1	AAAAAAAAAAAAAAA
MA0481.1	FOXP1	12.774	0.88055569921874	1100	1114	1	AAAAAAAAAAAAAAA
MA0481.1	FOXP1	12.774	0.88055569921874	1101	1115	1	AAAAAAAAAAAAAAA
MA0481.1	FOXP1	12.774	0.88055569921874	1102	1116	1	AAAAAAAAAAAAAAA
MA0481.1	FOXP1	12.774	0.88055569921874	1103	1117	1	AAAAAAAAAAAAAAA
MA0481.1	FOXP1	13.100	0.885897705609658	1104	1118	1	AAAAAAAAAAAAAAG
MA0481.1	FOXP1	9.600	0.828544876259308	1105	1119	1	AAAAAAAAAAAAAGA
MA0481.1	FOXP1	10.022	0.835459988826693	1106	1120	1	AAAAAAAAAAAAGAC
MA0481.1	FOXP1	7.985	0.802080642144789	1108	1122	1	AAAAAAAAAAGACCG

### 3.5 Gene expression profiling

**Figure 5:**
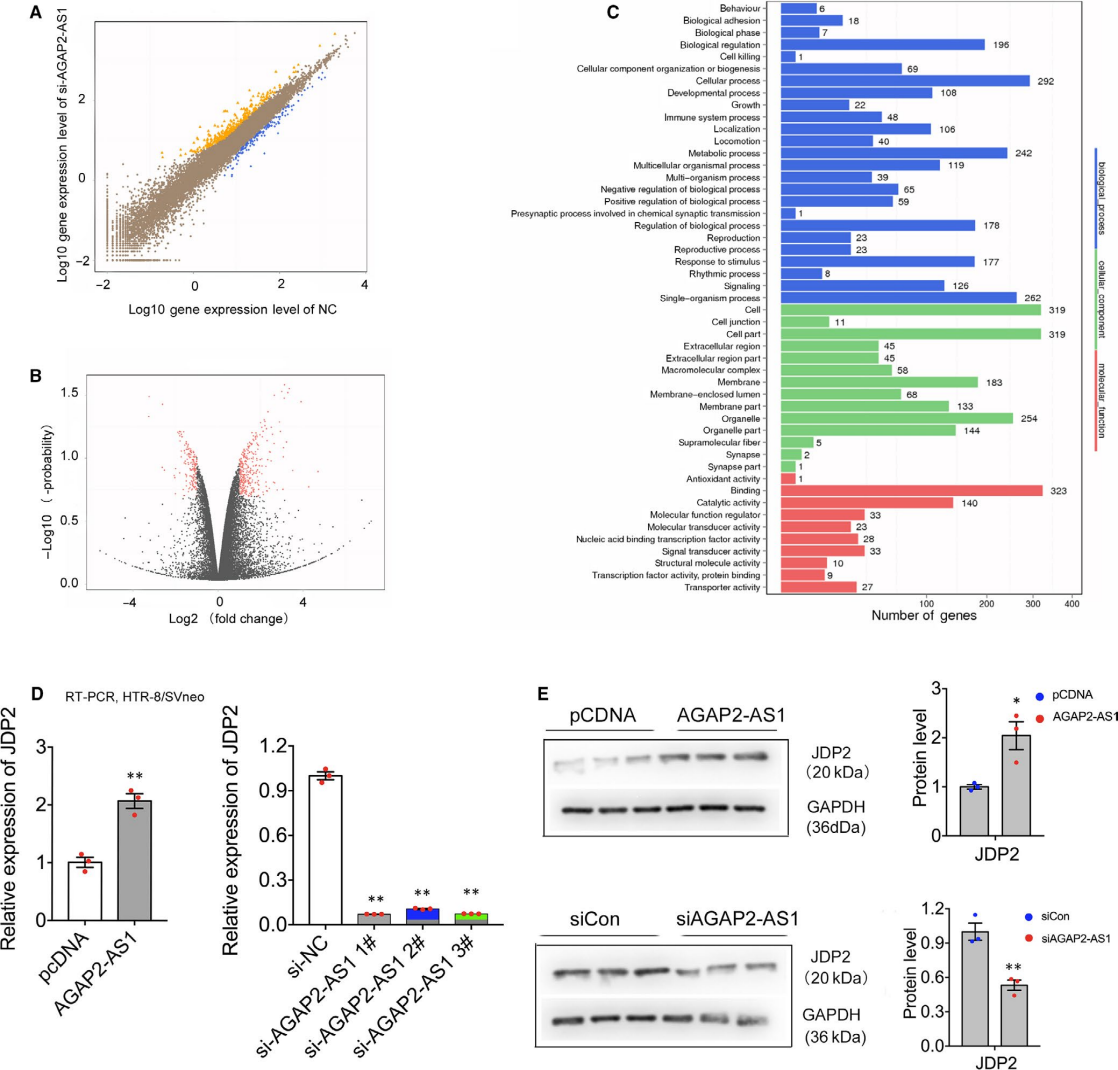
Gene Expression profiling. A and B, High‐throughput sequencing analysis was conducted to test downstream expression profiling after silencing of AGAP2‐AS1 in HTR‐8/SVneo. Orange represents upstream genes, blue represents downstream genes in A; red represents significantly change in B. C, GO analysis for all changed genes expression between siNC and siAGAP2‐AS1. (D and E) JDP2 expression level at protein and RNA level by Western blotting and qPCR, respectively, after transfected with AGAP2‐AS1 siRNAs in HTR‐8/SVneo. JDP2 expression level at protein and RNA level by Western blotting and qPCR, respectively, after transfected with AGAP2‐AS1 plasmid. All data are presented as the mean ± SEM, **P < .01, *P < .05

To clarify the molecular mechanism of AGAP2‐AS1–mediated regulation, the silencing of AGAP2‐AS1 was performed in human trophoblast HTR‐8/SVneo cells. After the cells were transfected with siRNAs against AGAP2‐AS1 for 48 hours, high‐throughput deep sequencing (RNA‐seq) analysis was performed. Based on the RNA‐seq results, the expression of 311 transcripts exhibited ≥twofold up‐regulation in the HTR/SVneo cells after the suppression of AGAP2‐AS1, whereas 152 transcripts exhibited ≤twofold down‐regulation (Figure 5A,B). The evaluation of the pathways activated by AGAP2‐AS1 after referencing the GO databases implied that cell migration and proliferation were associated with AGAP2‐AS1 expression in trophoblasts (Figure 5C). Subsequently, we performed real‐time PCR tests to verify whether many well‐known proliferation‐related and migration‐associated genes expressions, including TFPI2, RND3, HOXA7, KLF11, EGR1 and OSMR. were involved in the progression of PE (data not shown). Our results showed that Jun dimerization protein 2 (JDP2) levels were considerably decreased after the suppression of AGAP2‐AS1, whereas JDP2 levels were increased in the AGAP2‐AS1–depleted trophoblasts. This further suggests that the aberrantly expressed JDP2 protein may be a mediator of AGAP2‐AS1 suppression (Figure 5D). Subsequently, the results of Western blot analysis showed that the changes at the protein level were consistent with the mRNA results (Figure 5E; Tables S2 and S3).

### 3.6 AGAP2‐AS1 regulates JDP2 expression through the AGAP2‐AS1/miR‐574 axis

**Figure 6:**
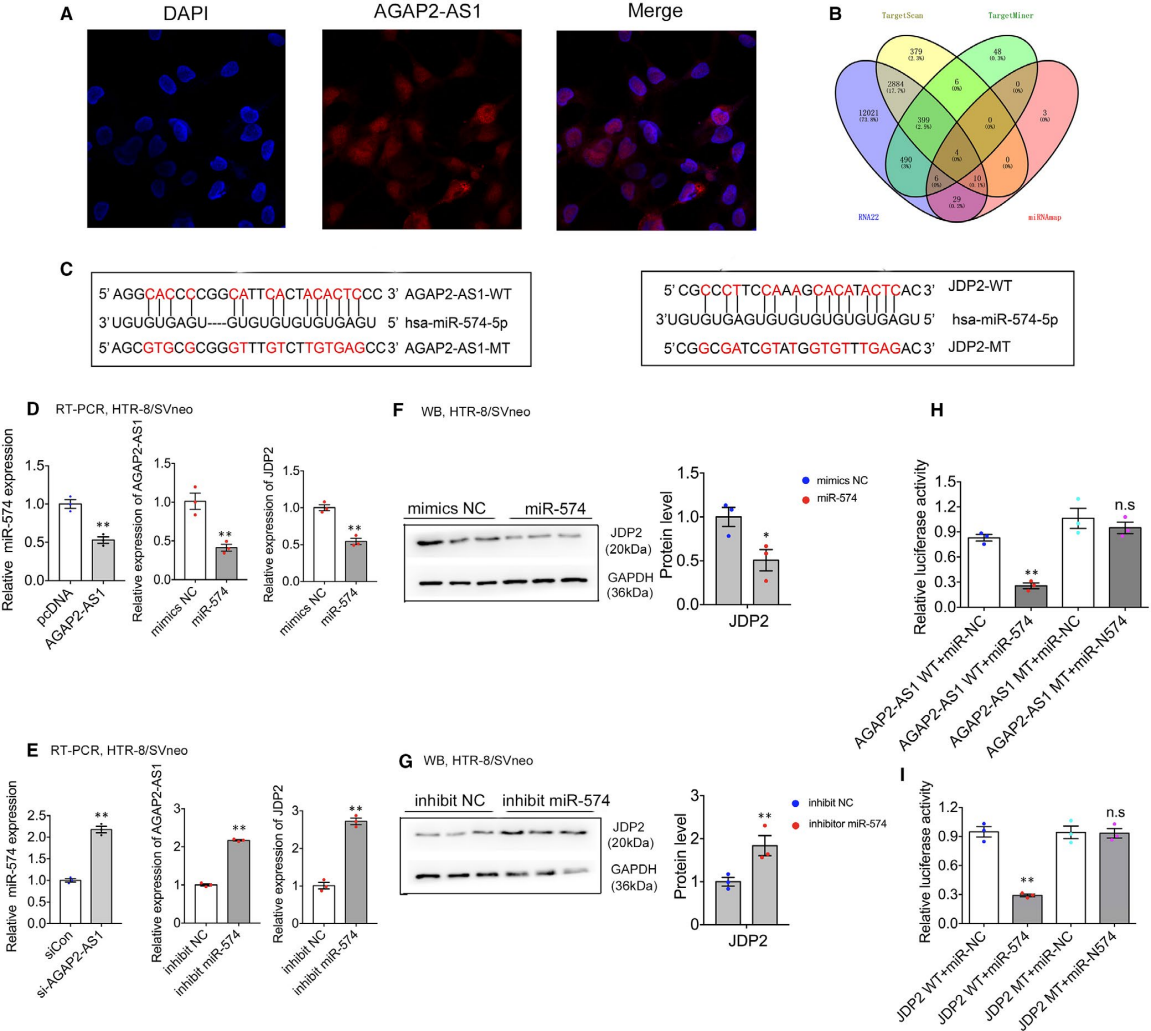
AGAP2‐AS1 regulates JDP2 expression by AGAP2‐AS1/miR‐574 axis. A, AGAP2‐AS1 location was detected by FISH in HTR‐8/SVneo. Red, AGAP2‐AS1; blue, DAPI. B, Bioinformatics analysis exhibited the binding probability between JDP2 and miR‐574. C, The RNAup algorithm predicted latent binding of miR‐574 to AGAP2‐AS1 and JDP2, with conservative sequence in the 3′‐UTR regions. D and E, HTR‐8/SVneo cells were treated with siCon or si‐AGAP2‐AS1, mimics NC or mimics miR‐574, inhibit NC or inhibitor miR‐574. Using RT‐PCR and Western blotting analysis to detect miR‐574 (on the left), AGAP2‐AS1 (in the middle) and JDP2 expression (on the right), respectively. F and G, Western blotting analysis was performed to test JDP2 expression at protein level after treated with mimics NC or mimics miR‐574, inhibit NC or inhibitor miR‐574. H, Luciferase reporter assays were conducted to detect the interacting activity between miR‐574 with AGAP2‐AS1/JDP2, normalized by Renilla activity. Numbers are shown as the mean ± SEM, **P < .01, *P < .05

To further explore the latent mechanisms of AGAP2‐AS1–mediated regulation in trophoblasts, we first conducted the fluorescence in situ hybridization (FISH) assays to test the subcellular localization of AGAP2‐AS1 in HTR‐8/SVneo. This suggested that AGAP2‐AS1 was localized in the nucleus as well as in the cytoplasm (Figure 6A). Recently, numerous studies have found a new lncRNA‐mediated regulatory pathway that can act as a ‘molecular sponge’ to bind microRNAs in the cytoplasm and subsequently modulate mRNAs at the post‐transcriptional level, further affecting cell biological function.^20^, ^21^ Based on the results of our FISH assay investigations, we suggested that AGAP2‐AS1 may function as a competing endogenous RNA to regulate JDP2 expression by competing for shared microRNAs in the HTR‐8/SVneo cells.

Next, we carried out bioinformatics analysis (TargetScan, TargetMiner, RNA22, miRNAmap) and predicted that miR‐574 can bind to the complementary sequences of both AGAP2‐AS1 and JDP2 (Figure 6B,C). Then, we conducted qRT‐PCR, and the results showed that silencing of AGAP2‐AS1 significantly up‐regulated the expression of miR‐574; in contrast, the miR‐574 level was decreased after AGAP2‐AS1 overexpression (Figure 6D,E). In addition, we performed RT‐PCR and Western blot assays to test the expression of AGAP2‐AS1 and JDP2 after the down‐regulation or up‐regulation of miR‐574. The results revealed that the inhibition of miR‐574 significantly up‐regulated AGAP2‐AS1 and JDP2 expression. In contrast, overexpression of miR‐574 could decrease AGAP2‐AS1 and JDP2 expression at both the RNA (Figure 6D,E) and protein levels (Figure 6F,G). Furthermore, we constructed diverse luciferase genes, including AGAP2‐AS1, mutant AGAP2‐AS1, 3’‐UTR of JDP2 and mutant 3’‐UTR of JDP2, cotransfected with miR‐574 mimics (Figure 6H,I). Through the construction of these diverse luciferase genes, we demonstrated that the relative luciferase activities of AGAP2‐AS1 and 3’ UTR of JDP2 were eliminated when the cells were transfected with the miR‐574 mimics. Conversely, there was no effect observed in either the mutant AGAP2‐AS1 or the mutant 3′UTR of JDP2 group. Thus, the resulting data validated the fact that miR‐574 can directly bind to both AGAP2‐AS1 and JDP2. Further, we demonstrated that AGAP2‐AS1 regulates JDP2 expression via the AGAP2‐AS1/miR‐574 axis in trophoblasts.

## 4 DISCUSSION

To date, there has been an abundance of evidence that highlights how the aberrant expression of lncRNAs could be involved in various human disorders, including PE.^22^ Our previous studies have shown that the down‐regulated expression of TUG1^7^ or HOXA11‐AS^10^ could significantly inhibit trophoblasts migration and proliferation and promote cell apoptosis in vitro. Additionally, other lncRNAs, such as HOTAIR,^23^ GHET1^24^ and TCL6,^25^ are reportedly able to regulate the trophoblastic phenotype that further accelerates the pathogenesis of PE. Thus, we need to identify more prospective PE‐related lncRNAs and further explore their biological functions and mechanisms in the context of PE.

In various reports, the association of AGAP2‐AS1 with numerous diseases, such as pancreatic cancer,^26^ glioblastoma^27^ and breast cancer^15^ has been identified. In this work, we found that lncRNA AGAP2‐AS1 is significantly down‐regulated in pre‐eclamptic placenta compared with that in the control group and that the lower expression of AGAP2‐AS1 is negatively correlated with the birth weight and gestational age of infants, indicating that down‐regulation of AGAP2‐AS1 is positively related to early termination of pregnancy, which further suggests that down‐regulation of AGAP2‐AS1 might lead to severe PE. We conducted specific functional assays to explore the potential implications of these findings. Up‐regulated AGAP2‐AS1 expression could promote trophoblast proliferation, invasion, and migration and inhibit cell apoptosis in trophoblasts. Conversely, the silencing of AGAP2‐AS1 stimulated cell apoptosis and reduced cell growth and invasion, thus establishing that the knockdown of AGAP2‐AS1 expression exerts inhibitory effects on trophoblasts by impairing the cellular phenotype, including cell growth, apoptosis and invasion. Thus, these results demonstrated that AGAP2‐AS1 might act as a molecular marker for the screening of PE.

Next, we performed the RNA‐seq (RNA transcriptome sequencing) assay to explore the potential downstream targets of AGAP2‐AS1. After the silencing of AGAP2‐AS1, we discovered a noticeable decrease in both the mRNA and protein levels of the cellular activator JDP2. JDP2, a member the activator protein family, has been implicated in many cellular processes including migration, invasion and proliferation.^28^, ^29^ For example, Yu W et al^30^ confirmed that down‐regulated expression of JDP2 can inhibit cell migration and invasion by promoting the epithelial‐mesenchymal transition in hepatocellular carcinoma. Moreover, Calicchio R et al^31^ has reported that JDP2 as a regulator of a subset of genes modified by pre‐eclamptic plasma. However, the roles of JDP2 in PE have not yet been investigated yet. Resultantly, there are still many gaps in the current understanding of JDP2 function and its roles in the biological mechanisms that result in PE. Further studies are needed to elucidate a JDP2‐associated mechanism that contributes to the pathogenesis of PE.

Based on the FISH assays and the bioinformatics analysis, which predicted that miR‐574 may be combined with the 3′‐UTR of both AGAP2‐AS1 and JDP2, we suspected that miR‐574 may exist as a key regulatory factor in the AGAP2‐AS1–mediated JDP2 up‐regulation network in trophoblasts. Thus, we performed real‐time PCR and Western blot investigations after silencing AGAP2‐AS1 or miR‐574, respectively. The results showed that, after inhibition of AGAP2‐AS1, miR‐574 expression was up‐regulated, whereas JDP2 expression was down‐regulated. Furthermore, we showed that AGAP2‐AS1 and JDP2 were significantly up‐regulated after silencing miR‐574. Subsequently, we conducted luciferase reporter assays and validated the fact that miR‐574 can directly bind to both AGAP2‐AS1 and JDP2 and demonstrated that AGAP2‐AS1 regulates JDP2 expression via the AGAP2‐AS1/miR‐574 axis in trophoblasts.

Recently, numerous studies have reported that transcription factors can regulate the activation or inactivation of lncRNA expression. For example, sp1 can induce lncRNA TINCR expression in lung adenocarcinoma^32^; and HOTAIR expression can be regulated by c‐Myc in gallbladder cancer.^33^ Our previous study has confirmed that AGAP2‐AS1 expression can be activated by the transcription factor RREB in pancreatic cancer.^26^ Nonetheless, in this study, we carried out bioinformatics analysis and found that FOXP1 may also induce AGAP2‐AS1 transcription. We conducted ChIP‐PCR to verify this, and the results showed that FOXP1 can directly bind to the promoter region of AGAP2‐AS1 in the HTR‐8/SVneo cells. In addition, qPCR data showed that the up‐regulated expression of FOXP1 can stimulate AGAP2‐AS1 transcription in trophoblasts. Overall, these results established the fact that AGAP2‐AS1 can be activated by FOXP1, further indicating that lncRNAs can be regulated simultaneously by many different transcriptional factors.

To conclude, in this report, we highlighted that the biological role and mechanism of AGAP2‐AS1 in PE are results of the down‐regulation of the expression of AGAP2‐AS1, which could inhibit trophoblast proliferation and invasion, and promote cell apoptosis through the suppression of JDP2 expression by sponging miR‐574 in the cytoplasm. These results reveal the obstacles of spiral artery remodelling, which further facilitate the occurrence and development of PE. This report enriched our understanding of the role of long non‐coding RNA in the pathogenesis of PE. However, in our study, other possible downstream and regulatory mechanisms that have the potential to be the biological phenotype of AGAP2‐AS1 were not explored. These are still not fully understood and need further investigation.

## CONFLICT OF INTEREST

None.

## AUTHOR CONTRIBUTIONS

Yetao Xu, Xi Xia and Sailan Wang carried out all experiments, analysed the data and prepared the pictures. Nana Yang and Ying Jiang collected the clinical data and sample tissues. Yuanyuan Zhang and Lizhou Sun conceived this research. Shilong Fu re‐checked and modulated the English for the current version. Yetao Xu and Dan Wu wrote this paper with the help of all the authors.

## Supporting information

Table S1

Table S2

Table S3

## Data Availability

All data generated or analysed during this study are included in this article.

## References

[1] Hofmeyr R , Matjila M , Dyer R . Preeclampsia in 2017: obstetric and anaesthesia management. Best Pract Res Clin Anaesthesiol. 2017;31:125‐138. 28625300 10.1016/j.bpa.2016.12.002

[2] Altman D , Carroli G , Duley L , et al. Do women with pre‐eclampsia, and their babies, benefit from magnesium sulphate? The Magpie Trial: a randomised placebo‐controlled trial. Lancet. 2002;359:1877‐1890. 12057549 10.1016/s0140-6736(02)08778-0

[3] Hogberg U . The World Health Report 2005: "make every mother and child count" ‐ including Africans. Scand J Public Health. 2005;33:409‐411. 16332605 10.1080/14034940500217037

[4] Phipps EA , Thadhani R , Benzing T , Karumanchi SA . Pre‐eclampsia: pathogenesis, novel diagnostics and therapies. Nat Rev Nephrol. 2019;15:275‐289. 30792480 10.1038/s41581-019-0119-6 PMC6472952

[5] Gyamfi‐Bannerman C , Pandita A , Miller EC , et al. Preeclampsia outcomes at delivery and race. J Matern Fetal Neonatal Med. 2019:1–8. 10.1080/14767058.2019.1581522 PMC6790144 30786794

[6] Marchese FP , Huarte M . Long non‐coding RNAs and chromatin modifiers: their place in the epigenetic code. Epigenetics. 2014;9:21‐26. 24335342 10.4161/epi.27472 PMC3928181

[7] Xu Y , Ge Z , Zhang E , et al. The lncRNA TUG1 modulates proliferation in trophoblast cells via epigenetic suppression of RND3. Cell Death Dis. 2017;8:e3104. 29022920 10.1038/cddis.2017.503 PMC5682669

[8] Wu D , Yang N , Xu Y , et al. lncRNA HIF1A antisense RNA 2 modulates trophoblast cell invasion and proliferation through upregulating PHLDA1 expression. Mol Ther Nucleic Acids. 2019;16:605‐615. 31085354 10.1016/j.omtn.2019.04.009 PMC6517652

[9] Xu Y , Lian Y , Zhang Y , et al. The long non‐coding RNA PVT1 represses ANGPTL4 transcription through binding with EZH2 in trophoblast cell. J Cell Mol Med. 2018;22:1272‐1282. 29193797 10.1111/jcmm.13405 PMC5783862

[10] Xu Y , Wu D , Liu J , et al. Downregulated lncRNA HOXA11‐AS affects trophoblast cell proliferation and migration by regulating RND3 and HOXA7 expression in PE. Mol Ther Nucleic Acids. 2018;12:195‐206. 30195759 10.1016/j.omtn.2018.05.007 PMC6023946

[11] Beckedorff F , Amaral M , Deocesano‐Pereira C , Verjovski‐Almeida S . Long non‐coding RNAs and their implications in cancer epigenetics. Biosci Rep. 2013;33. 10.1042/BSR20130054 PMC3759304 23875687

[12] Liu X , Chen H , Kong W , et al. Down‐regulated long non‐coding RNA‐ATB in preeclampsia and its effect on suppressing migration, proliferation, and tube formation of trophoblast cells. Placenta. 2017;49:80‐87. 28012459 10.1016/j.placenta.2016.12.004

[13] McAninch D , Roberts CT , Bianco‐Miotto T . Mechanistic insight into long noncoding RNAs and the placenta. Int J Mol Sci. 2017;18:1371. 10.3390/ijms18071371 PMC5535864 28653993

[14] Harati‐Sadegh M , Kohan L , Teimoori B , Salimi S . The long non‐coding RNA H19 rs217727 polymorphism is associated with PE susceptibility. J Cell Biochem. 2018;119:5473‐5480. 29380421 10.1002/jcb.26708

[15] Dong H , Wang W , Mo S , et al. SP1‐induced lncRNA AGAP2‐AS1 expression promotes chemoresistance of breast cancer by epigenetic regulation of MyD88. J Exp Clin Cancer Res. 2018;37:202. 30157918 10.1186/s13046-018-0875-3 PMC6114182

[16] Li W , Sun M , Zang C , et al. Upregulated long non‐coding RNA AGAP2‐AS1 represses LATS2 and KLF2 expression through interacting with EZH2 and LSD1 in non‐small‐cell lung cancer cells. Cell Death Dis. 2016;7:e2225. 27195672 10.1038/cddis.2016.126 PMC4917662

[17] Xu Y , Wu D , Jiang Z , et al. MiR‐616‐3p modulates cell proliferation and migration through targeting tissue factor pathway inhibitor 2 in preeclampsia. Cell Prolif. 2018;51:e12490. 30028057 10.1111/cpr.12490 PMC6528919

[18] Barsotti AM , Beckerman R , Laptenko O , et al. p53‐Dependent induction of PVT1 and miR‐1204. J Biol Chem. 2012;287:2509‐2519. 22110125 10.1074/jbc.M111.322875 PMC3268411

[19] Sato K , Nakagawa H , Tajima A , et al. ANRIL is implicated in the regulation of nucleus and potential transcriptional target of E2F1. Oncol Rep. 2010;24:701‐707. 20664976 10.3892/or_00000910

[20] Cesana M , Cacchiarelli D , Legnini I , et al. A long noncoding RNA controls muscle differentiation by functioning as a competing endogenous RNA. Cell. 2011;147:358‐369. 22000014 10.1016/j.cell.2011.09.028 PMC3234495

[21] Cao T , Jiang Y , Wang Z , et al. H19 lncRNA identified as a master regulator of genes that drive uterine leiomyomas. Oncogene. 2019;38:5356‐5366. 31089260 10.1038/s41388-019-0808-4 PMC6755985

[22] Gormley M , Ona K , Kapidzic M , et al. Preeclampsia: novel insights from global RNA profiling of trophoblast subpopulations. Am J Obstet Gynecol. 2017;217(200):e1‐e17. 10.1016/j.ajog.2017.03.017 28347715

[23] Mohammadpour‐Gharehbagh A , Jahantigh D , Saravani M , et al. Impact of HOTAIR variants on preeclampsia susceptibility based on blood and placenta and in silico analysis. IUBMB Life. 2019;71:1367‐1381. 31188529 10.1002/iub.2095

[24] Liu S , Xie X , Lei H , Zou B , Xie L . Identification of key circRNAs/lncRNAs/miRNAs/mRNAs and pathways in preeclampsia using bioinformatics analysis. Med Sci Monit. 2019;25:1679‐1693. 30833538 10.12659/MSM.912801 PMC6413561

[25] Wu JL , Wang YG , Gao GM , et al. Overexpression of lncRNA TCL6 promotes preeclampsia progression by regulating PTEN. Eur Rev Med Pharmacol Sci. 2019;23:4066‐4072. 31173275 10.26355/eurrev_201905_17907

[26] Hui B , Ji H , Xu Y , et al. RREB1‐induced upregulation of the lncRNA AGAP2‐AS1 regulates the proliferation and migration of pancreatic cancer partly through suppressing ANKRD1 and ANGPTL4. Cell Death Dis. 2019;10:207. 30814490 10.1038/s41419-019-1384-9 PMC6393474

[27] Luo W , Li X , Song Z , Zhu X , Zhao S . Long non‐coding RNA AGAP2‐AS1 exerts oncogenic properties in glioblastoma by epigenetically silencing TFPI2 through EZH2 and LSD1. Aging (Albany NY). 2019;11:3811‐3823. 31186379 10.18632/aging.102018 PMC6594811

[28] Yuanhong XU , Feng XU , Qingchang LI , Jianpeng F , Zhe L , Kejian G . Downregulation of AP‐1 repressor JDP2 is associated with tumor metastasis and poor prognosis in patients with pancreatic carcinoma. Int J Biol Markers. 2010;25:136‐140. 20677166 10.1177/172460081002500303

[29] Avraham S , Korin B , Aviram S , Shechter D , Shaked Y , Aronheim A . ATF3 and JDP2 deficiency in cancer associated fibroblasts promotes tumor growth via SDF‐1 transcription. Oncogene. 2019;38:3812‐3823. 30670778 10.1038/s41388-019-0692-y PMC6756089

[30] Yu W , Deng W , Zhao Q , Zhuang H , Zhang C , Jian Z . miR‐501 acts as an independent prognostic factor that promotes the epithelial‐mesenchymal transition through targeting JDP2 in hepatocellular carcinoma. Hum Cell. 2019;32:343‐351. 30877624 10.1007/s13577-019-00243-7

[31] Calicchio R , Buffat C , Mathieu JR , et al. Preeclamptic plasma induces transcription modifications involving the AP‐1 transcriptional regulator JDP2 in endothelial cells. Am J Pathol. 2013;183:1993‐2006. 24120378 10.1016/j.ajpath.2013.08.020

[32] Gao YW , Ma F , Xie YC , et al. Sp1‐induced upregulation of the long noncoding RNA TINCR inhibits cell migration and invasion by regulating miR‐107/miR‐1286 in lung adenocarcinoma. Am J Transl Res. 2019;11:4761‐4775. 31497197 PMC6731412

[33] Ma M‐Z , Li C‐X , Zhang Y , et al. Long non‐coding RNA HOTAIR, a c‐Myc activated driver of malignancy, negatively regulates miRNA‐130a in gallbladder cancer. Mol Cancer. 2014;13:156. 24953832 10.1186/1476-4598-13-156 PMC4085645

