# Supplementary material for: Down‐regulated lncRNA AGAP2‐AS1 contributes to pre‐eclampsia as a competing endogenous RNA for JDP2 by impairing trophoblastic phenotype
Source: J Cell Mol Med. 2020 Mar 9;24(8):4557–68. doi: 10.1111/jcmm.15113 (PMC7176850; doi:10.1111/jcmm.15113)
Supplement: Supplementary file 1 — Table S1 [file JCMM-24-4557-s001.docx]

| **Supplementary Table 1** |  |  | | |
| --- | --- | --- | --- | --- |
| **Real-time PCR primer sequences** | | |  |  |
| **Gene** | **Forward Primer** | **Reverse Primer** | | |
| GAPDH | 5’- CTTTGTCAAGCTCATTTCCTGG -3' | 5’- TCTTCCTCTTGTGCTCTTGC -3' | | |
| AGAP2-AS1 | 5’- TACCTTGACCTTGCTGCTCTC -3' | 5’- TGTCCCTTAATGACCCCATCC -3' | | |
| FOXP1 | 5’- GTTGCAGTCCTGTGGCATTA -3' | 5’- AGACCGCCGCACTCTAGTAA-3' | | |
| JDP2 | 5’- TGGGCTGTCTCTGTCTGTTG -3' | 5’- GCTCTGTCATCACTCAGGCA -3' | | |
| U6 | 5’- CTCGCTTCGGCAGCACA-3' | 5’- AACGCTTCACGAATTTGCGT-3' | | |
|  |  |  | | |
| **CHIP-PCR primer sequences** | | |  |  |
| AGAP2-AS1-P1 | 5’- AACCCCAGTACCCCATCTTG -3' | 5’- GGTAATTGGGGAGGGGAGAG -3' | | |
|  |  |  | | |
| **FXOP1 siRNA sequence (human)** | | |  |  |
| **1# sense 5’- CTCAGTCCACACTCCCAAA -3’** | | |  |  |
| **2# sense 5’- CCACAGAGCTTACCTCATA -3’** | | |  |  |
| **3# sense 5’- CTGGTTCACACGAATGTTT -3’** | | |  |  |
|  | | |  |  |
| **AGAP2-AS1 siRNA sequence (human)** | | |  |  |
| **1# sense 5’- ACCGUCUCAGGUUCGCACACCCUCA-3’** | | |  |  |
| **2# sense 5’- UCUGUUCCCUCACGCUUACCGCGAA-3’** | | |  |  |
| **3# sense 5’- CGCAGCUGCAGAGAGGGUUUGGGUU-3’** | | |  |  |
|  | | |  |  |
| **AGAP2-AS1 probe sequences** | | |  |  |
| **5’- CTTGGAGGCAGAGTCAGTGCCAACCCAAACC- 3’** | | |  |  |
|  | | |  |  |
|  | | |  |  |
|  | | |  |  |
